# From Assistance to Autonomy: Evaluating Procedural Competency in Pediatric Emergency Medicine

**DOI:** 10.1002/aet2.70095

**Published:** 2025-09-28

**Authors:** Richard Barber, Marideth Rus, Elizabeth Moran, Esther M. Sampayo, Deborah Hsu, Corrie E. Chumpitazi, Elizabeth A. Camp, Nidhi V. Singh

**Affiliations:** ^1^ Division of Pediatric Emergency Medicine, Department of Pediatrics Baylor College of Medicine Houston Texas USA; ^2^ Division of Pediatric Emergency Medicine, Department of Emergency Medicine and Pediatrics Stanford University Stanford California USA; ^3^ Division of Emergency Medicine, Department of Pediatrics Duke University Durham North Carolina USA

**Keywords:** evaluation, fellows, procedure

## Abstract

**Objective:**

Pediatric emergency medicine (PEM) physicians require expertise in numerous procedural skills to manage emergencies in children. Fellows require hands‐on experience, expert supervision, and standardized feedback to build procedural competency. Our objective was to develop and gather validity evidence for an evaluation tool to assess PEM fellows' ability to perform procedures.

**Methods:**

We conducted a retrospective study of PEM fellows' procedural performance within a children's hospital system. Faculty evaluated fellows using a one‐item, five‐point entrustment/level of supervision scale. We focused on three frequently performed procedures: laceration repair, intubation, and procedural sedation. To assess changes in supervision scores by training year, we used a mixed‐effects binary logistic regression model with scores ≥ 4 as the threshold. Adjusted odds ratios (aOR), 95% confidence intervals (CIs) and *p* values were reported. We also assessed inter‐ and intra‐rater reliability overall, by procedure, and by semester.

**Results:**

Data from 38 fellows and 137 supervising faculty evaluations were included. Compared to first‐year fellows, second‐year fellows were significantly more likely to receive higher scores (aOR = 4.95; 95% CI 3.78–6.47) and third‐year fellows even more so (aOR = 10.15; 95% CI 6.71–15.35). Intra‐rater reliability showed moderate to very strong correlation (*ρ* = 0.84), and by procedure: intubation (*ρ* = 0.78), lac repair (*ρ* = 0.92), and sedation (*ρ* = 0.85). Inter‐rater reliability was poor across all measures. First‐year fellows showed significant differences in intubation scores between specialties. No significant differences were found among third‐year fellows.

**Conclusion:**

A supervision‐based evaluation tool demonstrated strong intra‐rater reliability and captured increasing procedural competency among PEM fellows, as evidenced by progressively higher entrustment scores with increasing years in training. This tool may support standardized assessment and meaningful feedback throughout fellowship training.

## 1 Background

A pediatric emergency medicine (PEM) physician requires expertise in numerous procedural skills to manage medical and surgical emergencies in children effectively. The Accreditation Council for Graduate Medical Education (ACGME) has published a list of essential procedures identified by expert consensus for PEM fellowship graduates in the United States (US) [1]. Demonstrating “competence in performing common procedures” has been identified by the American Board of Pediatrics as an entrustable professional activity (EPA) for pediatric emergency medicine physicians [2]. An EPA is a “unit of professional practice that can be fully entrusted to a learner, once they have demonstrated competence to execute the task unsupervised.” [3] As medical education continues its transition to a competency‐ and outcomes‐ based system that utilizes the framework of EPAs, workplace‐ and EPA‐based assessments, standardized for use across institutions, are needed [4, 5, 6].

Specific to developing procedural competency, trainees require hands‐on experience, expert supervision, accurate evaluations, and constructive feedback [7]. A 2023 study evaluating the use of a three‐tier level of supervision scale provided internal medicine residents meaningful feedback about their procedural competence and helped guide program development [7]. The Direct Observation of Procedural Skills (DOPS) tool utilizing supervision level ratings ranging from “unable to perform the procedure under supervision” to “competent to perform the procedure unsupervised” has been adopted by surgical training programs to evaluate residents across specific procedural domains [7]. For pediatric subspecialty fellows, previous studies have demonstrated validity evidence in using entrustment/level of supervision ratings to evaluate fellows' performance of common and subspecialty‐specific EPAs [8, 9, 10].

The primary objective of our study was to develop and gather validity evidence for a workplace‐based assessment tool utilizing an entrustment/level of supervision scale for supervising faculty physicians to evaluate pediatric emergency medicine fellows' ability to perform procedures. Our secondary objective was to describe the reliability of the tool for use across different clinical settings by various supervising physicians.

## 2 Methods

This study was approved by the local IRB.

### 2.1 Study Setting and Population

We conducted a retrospective, cross‐sectional study of PEM fellows' procedural performance at two freestanding children's hospitals within the same hospital system. One is a level 1 trauma center; the other is a level 4 trauma center. The two emergency departments (EDs) had a combined annual patient visits of 117,434 in 2024. The PEM fellowship program, one of the largest in the US, enrolls six fellows annually, with 18–19 fellows per year. Board‐certified PEM physicians supervise procedures in the ED, while attending physicians with board certifications in other subspecialties like pediatric anesthesiology supervise procedures fellows perform outside of the ED.

### 2.2 Study Instrument

The one‐item assessment tool initially developed to assess trainees utilized the ABP‐suggested entrustment/level of supervision scale for the PEM subspecialty‐specific procedural EPA [1]. This tool was reviewed by a panel of PEM faculty with education expertise, including three current and former PEM fellowship program directors, associate PEM fellowship directors, resident education directors, as well as faculty who would serve as end users of the instrument and PEM fellows who would be evaluated with the instrument. Minor syntax modifications were made to the ABP‐suggested rating scale to address concerns for wording ambiguity. The concept of rating fellows utilizing levels of supervision was kept intact. The tool was piloted during the academic year prior to study implementation, with further minor adjustments made to scale verbiage. These steps helped to support content validity and feasibility of the study instrument.

### 2.3 Instrument Implementation

Instructions for use of the evaluation tool were provided during PEM faculty division meetings, focused training of core faculty during quarterly division educator meetings, and email communications to PEM faculty and Pediatric Anesthesiology rotation directors who would be evaluating PEM fellows using this tool. PEM faculty utilized the study tool (Table 1) to evaluate fellows on specific procedures they performed in the ED. Pediatric Anesthesiologists used the same scale to evaluate first and third‐year PEM fellows' performance of intubations in the operating room during fellows' anesthesia rotations.

**TABLE 1:** Entrustment scale.

Scale	Level of supervision
1	Trusted to observe and/or participate
2	Trusted to execute with direct supervision and coaching
3	Trusted to execute with indirect supervision for simple cases only; complex cases require direct supervision
4	Trusted to execute with indirect supervision for most cases; select complex cases require discussion and/or direct supervision at critical portions
5	Trusted to execute independently without supervision

### 2.4 Data Collection

Fellows logged completed procedures on MedHub with the following data included: Procedure performed, supervising physician's name, date procedure performed, and patient demographics. MedHub autogenerated two evaluation requests to the supervising physician to verify completion of the procedure by the PEM fellow and evaluate the fellow's performance using the study tool, which were sent immediately upon the fellow logging the procedure. The date the fellow logged the procedure on MedHub and the date when the faculty evaluator submitted their evaluation were also collected. Study investigators were provided de‐identified data of all evaluations collected between July 2019 and June 2024. Evaluations for procedural sedation, laceration repair, and intubation were included; evaluations for other procedures were excluded.

### 2.5 Data Analysis

We selected three procedures (procedural sedation, laceration repair, and intubation) to evaluate based on a 2020 study identifying them as the most frequently performed in US‐based PEM fellowship programs [11].

A high entrustment/level of supervision score was defined as a 4 or 5 rating. A rating of 4 entrusts the fellow to perform the procedure with indirect supervision (supervisor not in room) for most cases, with select complex cases requiring discussion. A score of 5 entrusts the fellow to perform the procedure independently without supervision. This decision to include 4 and 5 ratings as high ratings aligns with other entrustment‐based frameworks where intermediate to advanced levels of supervision imply readiness for unsupervised or minimally supervised practice [12]. A 2022 study found that most PEM fellowship program directors reported they would graduate PEM fellows assessed to be trusted to perform the six PEM EPAs with indirect supervision or with discussion and direct supervision for critical or complex patient cases [12].

**FIGURE 1:**
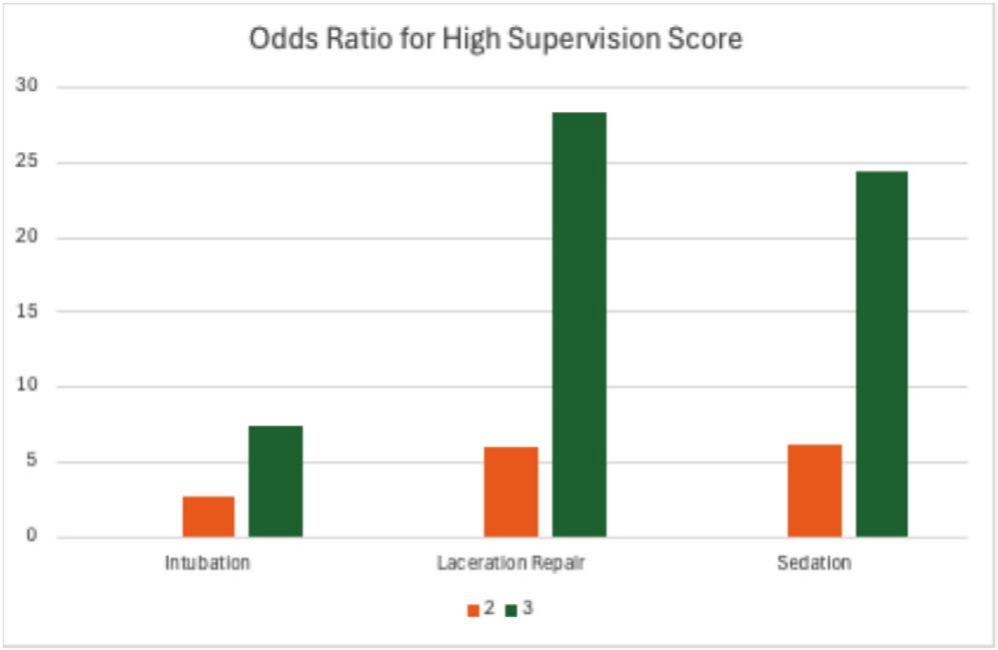
Adjusted odds ratio of receiving a high supervision score (4 or 5) over time for each procedure with first year as the reference and adjusted for evaluator subspecialty.

Descriptive statistics summarized the study population. To assess the primary objective, a mixed‐effects binary logistic regression model, treating fellow ID as a random effect, was utilized to analyze program year (1–3) and high supervision score (four or greater). Effect estimates were adjusted for semester/year, evaluators' subspecialty, and procedure type. Adjusted odds ratios (aOR), 95% confidence intervals (CIs) and *p* values were reported. Additionally, program year and high evaluation scores were stratified by procedure type. Using a mixed‐effects binary logistic regression model, with fellow ID set as a random effect and adjusted for evaluators' subspecialty, aORs and 95% CIs were graphed using a bar graph (see Figure 1).

For reliability analyses, we assessed consistency: overall, by procedure and by year/semester year. We calculated inter‐rater reliability (different evaluators assessing the same fellow) using Cronbach's alpha, reporting medians and interquartile ranges (IQR). Values > 0.7 were deemed acceptable, > 0.8 good, and > 0.9 excellent. Intra‐rater reliability (same evaluator assessing the same fellow over time) was assessed with Spearman's rho test, along with the median and IQR. The Spearman's rho was interpreted as 0.3–0.5 as fair, 0.6–0.7 as moderate, and 0.8–0.9 as very strong. Reliability analyses were based on fellow ID, semester/year, procedure type, and supervisor ID. We compared the first and last evaluations within each semester/year and procedure. One sample for each fellow was taken: one for different evaluators and one for same evaluators.

For the subgroup analysis, we compared anesthesiology and PEM faculty evaluation scores for first‐ and third‐year fellows on intubation using both t‐tests (means, SD) and Mann–Whitney tests (median, IQRs). A *p* value < 0.05 indicated significance. All analyses were conducted using STATA, version 13.1 (StataCorp LP, College Station, TX).

## 3 Results

Data from 38 fellows and 137 supervising faculty evaluations were included (Table 2). 57% of supervising physicians were PEM subspecialists, with the remaining 43% being pediatric anesthesiologists (Table 2). Compared to first‐year fellows, second‐year fellows were significantly more likely to receive higher scores (aOR = 4.95; 95% CI 3.78–6.47) and third‐year fellows even more so (aOR = 10.15; 95% CI 6.71–15.35) (Table 3). Stratified models showed increased aORs by year for all procedures (Figure 1). The median time from fellows' logging a procedure to faculty submitting the evaluation was 12 days (IQR 1–37 days).

**TABLE 2:** Description of study population (*N* = 2799).

	*N* (%)
Number of Fellows (based on ID)	38
Program year	
1	1372 (49.02)
2	917 (32.76)
3	510 (18.22)
Number of procedure types	
Intubation	1157 (41.34)
Laceration Repair	758 (27.08)
Sedation	884 (31.58)
Number of evaluators (based on ID)	137
Evaluator's subspecialty	
PEM	78 (56.93)
Anesthesia	59 (43.07)

**TABLE 3:** Adjusted odds ratio of high supervision score (four or higher) and training year (*N* = 2799).

Program year	aOR (95% CI)^a^	*p* value
1	Ref	
2	4.95 (3.78–6.47)	< 0.001
3	10.15 (6.71–15.35)	< 0.001

Abbreviations: aOR, adjusted odds ratio; CI, confidence interval; Ref, reference.

^a^ Adjusted for semester/year, evaluator subspecialty, and procedure type. The fellow ID was set as a random effect.

The reliability analysis ranged from less than acceptable to very strong. Inter‐rater reliability was poor overall (Cronbach's alpha = 0.4), which was consistent by procedure type and by semester year (Table 4). In contrast, intra‐rater reliability showed moderate to very strong correlation: overall *ρ* = 0.84; intubation = 0.78, lac repair = 0.92, and sedation = 0.85; and semester/year (range 0.64–0.92) (Table 5).

**TABLE 4:** Inter‐rater reliability (different raters/ordinal data) (*N* = 304).

	N of pairs	Cronbach's alpha	Evaluators score 1 median (IQR)	Evaluators score 2 median (IQR)
Overall	304	0.40	4 (3, 4)	4 (3, 5)
Intubation	110	0.41	3 (3, 4)	4 (3, 5)
Laceration repair	91	0.29	4 (3, 4)	4 (3, 5)
Sedation	103	0.39	4 (3, 5)	4 (4, 5)
Fall 2019	17	0.01	4 (2, 4)	4 (3, 4)
Spring 2020	24	0.47	3 (3, 5)	4 (3, 4)
Fall 2020	35	0.29	4 (3, 5)	4 (4, 5)
Spring 2021	29	0.19	4 (3, 5)	4 (4, 5)
Fall 2021	35	0.66	4 (3, 4)	4 (3, 5)
Spring 2022	32	0.48	4 (3, 4)	4 (3, 5)
Fall 2022	42	0.32	4 (3, 5)	4 (3, 5)
Spring 2023	33	0.57	3 (3, 4)	4 (3, 4)
Fall 2023	32	0.06	3 (3, 4)	4 (4, 5)
Spring 2024	25	0.44	4 (3, 4)	4 (4, 5)

**TABLE 5:** Intra‐rater reliability (same raters/ordinal data) (*N* = 183).

	N of pairs	Spearman's rho	Evaluators first score median (IQR)	Evaluators second score median (IQR)
Overall	183	0.84	4 (3, 5)	4 (3, 5)
Intubation	68	0.78	4 (3, 4)	4 (3, 4)
Laceration repair	49	0.92	4 (3, 5)	4 (4, 4)
Sedation	66	0.85	4 (3, 5)	4 (4, 5)
Fall 2019	9	0.90	4 (4, 5)	4 (4, 4)
Spring 2020	15	0.85	3 (3, 5)	4 (3, 5)
Fall 2020	23	0.92	4 (3, 5)	4 (3, 5)
Spring 2021	14	0.89	4 (4, 5)	4.5 (4, 5)
Fall 2021	23	0.87	4 (3, 5)	4 (3, 5)
Spring 2022	22	0.78	4 (3, 4)	4 (4, 4)
Fall 2022	24	0.65	4 (3.5, 4.5)	4 (3, 4)
Spring 2023	18	0.89	4 (3, 4)	4 (3, 5)
Fall 2023	22	0.92	4 (3, 5)	4 (4, 5)
Spring 2024	13	0.64	4 (3, 5)	4 (4, 5)

Ratings of first year fellows' intubations by PEM faculty were significantly higher than ratings by Pediatric Anesthesiology faculty (Table 6). No significant differences in ratings of third year fellows' intubations were found between these subspecialty attending groups (Table 6).

**TABLE 6:** Mean and Median evaluation scores of first‐ and third‐year fellows comparing evaluation scores between Anesthesia and PEM faculty for endotracheal intubation.

First year fellows' evaluation scores				
Procedure	Faculty	*N*	Mean (± SD)	*p* value
Intubation	Anesthesia	223	3.37 (0.98)	0.01
	PEM	284	3.60 (0.89)	

		*N*	Median (IQR)	*p* value
Intubation	Anesthesia	223	3 (3.4)	0.01
	PEM	284	4 (3.4)	

Third Year Fellows' Evaluation Scores				
Procedure	Faculty	*N*	Mean (± SD)	*p* value
Intubation	Anesthesia	119	4.37 (3.45)	0.37
	PEM	136	4.10 (0.75)	

		*N*	Median (IQR)	*p* value
Intubation	Anesthesia	119	4 (4.4)	0.57
	PEM	136	4 (4.5)	

## 4 Discussion

We utilized and evaluated an entrustment/level of supervision scale to assess PEM fellows' procedural performance for laceration repair, endotracheal intubation, and procedural sedation. Unlike prior studies that rely on self‐report, we developed an evaluation tool utilized by faculty to evaluate fellows for each procedure they performed [13, 14]. We demonstrated fellows' increasing entrustability in performing these three procedures over time. When using first‐year fellows as a reference, odds of receiving a high score increased significantly in the second and third years. Our results align with expectations for skill progression in performing procedures as has been demonstrated in other studies in which level of supervision scales were used to evaluate internal medicine and surgery residents as well as pediatric subspecialty fellows including pediatric emergency medicine fellows [7, 8, 9, 10]. The simple and intuitive instrument we developed and utilized in our study may allow PEM fellowship programs to provide structured feedback throughout training to PEM fellows, facilitate their ability to perform procedures without supervision, and allow programs to demonstrate trainee competence in this crucial aspect of PEM practice.

Our study findings provide further support for use of EPA and workplace‐based assessments utilizing entrustment/level of supervision rating scales. EPAs provide a shared framework that is intuitive to both trainees and faculty because this framework mirrors training processes that naturally occur in clinical learning environments [4, 5, 6]. The general concept of competency‐based medical education (CBME) requires empirical and conceptual support, and the use of EPAs can serve as an effective framework for CBME programs [4, 5, 6]. Using EPAs as a competency framework for education and assessment focuses on gradual autonomy that happens naturally in the clinical setting and breaks down summative entrustment into relevant units for professional practice [4, 5, 6]. While traditional assessments of competence have involved regarding trainee performance retrospectively, entrustment decision‐making asks the supervisor to use their expertise to make judgments about readiness for future performance [4, 5, 6]. In our study, supervising physicians observed PEM fellows each time they performed one procedure and provided an assessment of their entrustability to perform future procedures. Our study contributes to the body of literature that can inform programmatic transition to CBME.

Anesthesiology faculty evaluated PEM fellows on intubation procedures performed during their anesthesiology rotations in the first and third year of training. First‐year fellows received higher ratings from PEM faculty than from anesthesiology faculty. In the ED, PEM physicians supervise emergent intubations on critically ill patients where conditions are unpredictable and time‐sensitive. In contrast, anesthesiology faculty supervise trainee intubations of patients during scheduled surgeries in the operating room, an environment that is considered more controlled with stable patients. Additional ED‐specific challenges include critically ill patients being less tolerant of prolonged and failed intubation attempts, changing dynamics of care teams, and the unplanned nature of patient presentations [15]. We postulate that the difference in ratings between anesthesiology and PEM faculty for this group of fellows is due to PEM faculty observing first‐year fellows performing intubations in the ED after they complete their anesthesiology rotations. In our institution, first‐year fellows may be complete novices in performing intubations at the start of their fellowship. They do not routinely intubate patients in the ED until after their anesthesiology rotations. As such, fellows in their first‐year anesthesiology rotations are being evaluated by anesthesiology faculty during the time when they are least experienced with intubations. By the time they are allowed to intubate in the ED, they would presumably have gained some competency in performing intubations. Despite the less controlled setting and more critical nature of patients requiring emergent intubations in the ED, fellows should be able to demonstrate, at the very least, rudimentary skills necessary to intubate ED patients compared to potentially lacking even the most basic skills during their first‐year anesthesiology rotation.

No significant difference in intubation supervision scores was observed between PEM and anesthesia faculty evaluations of third‐year fellows. Our finding shows that fellows attain increased ratings with progression through training as evaluated by two different sets of evaluators, suggesting the development of competence in performing intubations during fellowship. A 2024 study using video recordings of PEM fellows performing intubations in the trauma bay over a 6‐year study period found that no PEM fellow achieved a 90% success rate for first or second attempts, and a minority reached 80%. This study also found no correlation between increasing training duration and intubation success rates [15]. The differences between our study findings and those of this study may suggest that various factors outside of physician competence and ability to demonstrate necessary skills to intubate patients affect intubation success rates. Such factors may include airway complexity, equipment malfunction, unplanned events during intubation, and infrequency of practice leading to skills decay in the performance of high‐acuity procedures that occur infrequently, resulting in low opportunities for routine practice.

To evaluate the internal structure of our study instrument, we compared evaluations by different faculty evaluating the same fellow (inter‐rater reliability) versus evaluations from a single faculty for the same fellow (intra‐rater reliability) during the study period. Inter‐rater reliability was low. We hypothesize that this is due to a large number of faculty who individually may have limited opportunities to work with fellows and may not be uniformly familiar with providing evaluations, inconsistent faculty training on utilizing the evaluation tool, and varied levels of comfort with granting autonomy. Conversely, intra‐rater reliability was moderate to very strong: intubation (*ρ* = 0.78), procedural sedation (*ρ* = 0.85), and laceration repair (*ρ* = 0.92). This strong intra‐rater reliability may reflect that individual faculty, once familiar with the evaluation tool and their own expectations for fellow performance, apply the supervision scale consistently across time, even with the complexity and variability of the patients in the ED. Faculty also have subjectivity in their grading and may demonstrate consistency in that subjectivity, with some faculty being considered “easy graders” and others “hard graders”. Rater idiosyncrasy in workplace‐based assessment can make it challenging to obtain accurate and reliable performance evaluations, as the variability in performance ratings stems from individual differences among raters rather than differences in trainees' actual performance [16]. The moderate intra‐rater reliability found in intubation evaluations may reflect its diverse clinical contexts and complexity. The high reliability for sedation may stem from institutionally standardized protocols, leading evaluators to expect consistent fellow performance. The strong intra‐rater reliability for laceration repairs likely reflects the routine and standardized nature of repairing pediatric lacerations. Patients with lacerations present frequently to emergency departments, accounting for up to 8.2% of annual ED visits [17]. Most pediatric lacerations performed by PEM physicians are considered simple, requiring one layer closure. Lacerations that require multiple layer closures, involve more sensitive areas, and/or procedural sedation to be performed simultaneously for successful wound closure are more likely to be completed in the ED by surgical consultants [18].

While our study was conducted at a large, high‐volume level 1 pediatric trauma center with frequent procedural opportunities, we recognize that there is variation in the frequency of clinical exposures and different supervisory structures at various institutions. Investigation of the feasibility and utility of our evaluation tool for use at other institutions is needed. However, the conceptual framework—assessing the level of supervision needed and entrustability in real time—remains applicable across different clinical settings and may be useful for identifying issues related to procedural opportunities for trainees.

There were several limitations of this study. Third‐year fellows logged fewer procedures relative to first‐and second‐year fellows. Possible reasons for this finding include fellows being given graduated autonomy leading to more indirect as opposed to direct supervision of procedures, making it difficult for faculty to evaluate procedural performance; decreased fellows' motivation to log familiar procedures; and reduced clinical time, with a significant portion of fellowship time allocated to scholarship during the final year of training. In some cases, late submission of evaluations by faculty could have led to recall bias. There were also some instances of faculty verifying performance of procedures without submitting evaluations of the fellows' performance, leading to loss of data points. In order to mitigate recall bias, the amount of time faculty have to complete evaluations can be limited to 7 days or less, and a process to incentivize faculty submission of evaluations may lead to more consistent submission of evaluations. Fellows may have also delayed logging procedures, further widening the timing gap to evaluation and decreasing the accuracy of evaluator recall. There was a lack of availability of additional data points on outcomes to support procedural success, such as the number of attempts or complications. We also recognize that the decision to define a score of 4 or 5 as competency is somewhat subjective. Finally, our inability to attain 100% capture for formal calibration and structured orientation of supervising faculty in the use of the evaluation tool likely contributed to variability in scoring.

Future effort is needed to improve fellow reporting of procedures and improve inter‐rater reliability of this evaluation tool. Incorporation of training for supervising physicians that aligns with established standards of simulation assessment best practices, including frame‐of‐reference training, may be a strategy to consider [19]. Including a comment section in the assessment tool may allow provision of procedure‐specific formative feedback to trainees that could lead to further improvement of skills and higher entrustability ratings for future procedures. Including additional definitions of procedural competence such as number of attempts needed to complete a procedure, complications, or patient satisfaction may also be helpful.

## 5 Conclusion

We provide validity evidence for a workplace‐based one item assessment utilizing an entrustment/level of supervision scale to assess pediatric emergency medicine fellows on the entrustable professional activity of performing common procedures. The procedures specifically studied in this project were procedural sedation, endotracheal intubation, and laceration repair. Fellows received increasing entrustability ratings as they progressed through training years. The evaluation tool demonstrated strong intra‐rater reliability. This method of assessment may contribute to the future standardization of evaluating procedural competency of pediatric emergency medicine fellows across institutions.

## Author Contributions

R.B.B.: conceptualized and designed the study, designed the data collection instruments, collected data, interpreted study results, drafted the initial manuscript, and reviewed and revised the manuscript. D.H., M.R., E.M., C.C., E.M.S., and N.V.S.: conceptualized and designed the study, designed the data collection instruments, interpreted study results, and performed critical revision of the manuscript for important intellectual content and statistical expertise. E.A.C. interpreted data results and reviewed and revised the manuscript for statistical expertise. All authors approved the final manuscript as submitted and agreed to be accountable for all aspects of the work.

## Conflicts of Interest

The authors declare no conflicts of interest.

## Data Availability

The data that support the findings of this study are available from the corresponding author upon reasonable request.
